# Early Surfactant Therapy for Respiratory Distress Syndrome in Very Preterm Infants

**DOI:** 10.3390/healthcare11030439

**Published:** 2023-02-03

**Authors:** Manuela Cucerea, Mihaela Moscalu, Elena Moldovan, Reka Santa, Zsuzsanna Gall, Laura Mihaela Suciu, Marta Simon

**Affiliations:** 1 Department of Neonatology, George Emil Palade University of Medicine, Pharmacy, Science, and Technology, 540142 Targu Mures, Romania; 2 Department of Preventive Medicine and Interdisciplinarity, “Grigore T. Popa” University of Medicine and Pharmacy, 700115 Iași, Romania; 3 Pediatric Intensive Care Unit, Cardiovascular and Transplant Emergency Institute, 540136 Targu-Mures, Romania

**Keywords:** LISA, INSURE, surfactant, respiratory distress syndrome, preterm infant

## Abstract

Background: It is currently considered that early initiation of nasal continuous positive airway pressure, using a less invasive exogenous surfactant administration and avoiding mechanical ventilation as much as possible to minimize lung damage, may reduce mortality and/or the risk of morbidities in preterm infants. The aim of our study was to quantify our experience and compare different strategies of surfactant administration, to investigate which method is associated with less morbidity. Materials and Methods: A total of 135 preterm infants with early rescue surfactant administration for respiratory distress syndrome were included in the study. The infants were treated in an academic, Level III Neonatal Intensive Care Unit over a 3-year period between 1 December 2018 and 1 December 2021. Patients were separated into three groups: those with standard surfactant administration; those with Less Invasive Surfactant Administration—LISA; and those with Intubation Surfactant Administration Extubation—INSURE. As a primary outcome, we followed the need for intubation and mechanical ventilation within 72 h, while the secondary outcomes were major neonatal morbidities and death before discharge. Results: The surfactant administration method was significantly associated with the need for mechanical ventilation within 72 h after the procedure (*p* < 0.001). LISA group infants needed less MV (OR = 0.538, *p* = 0.019) than INSURE group infants. We found less morbidities (OR = 0.492, *p* = 0.015) and deaths before discharge (OR = 0.640, *p* = 0.035) in the LISA group compared with the INSURE group. The analysis of morbidities found in infants who were given the surfactant by the LISA method compared with the INSURE method showed lower incidence of pneumothorax (3.9% vs. 8.8%), intraventricular hemorrhage (17.3% vs. 23.5%), intraventricular hemorrhage grade 3 and 4 (3.9% vs. 5.9%), sepsis/probable sepsis (11.5% vs. 17.7%) retinopathy of prematurity (16.7% vs. 26.7%) and deaths (3.9% vs. 5.9%). There were no significant differences between groups in frequencies of bronchopulmonary dysplasia, necrotizing enterocolitis and patent ductus arteriosus. Conclusions: Less invasive surfactant administration methods seem to have advantages regarding early need for mechanical ventilation, decreasing morbidities and death rate. In our opinion, the LISA procedure may be a good choice in spontaneously breathing infants regardless of gestational age.

## 1. Introduction

Once the etiology of respiratory distress syndrome (RDS) was discovered, exogenous surfactant administration became the most effective evidence-based therapy for RDS in preterm infants [1]. Despite the benefits of surfactant administration, complications may also occur following this procedure depending on the administration method that is used. Occasionally, endotracheal intubation may cause hypoxia, bradycardia or respiratory tract trauma [2,3]. Mechanical ventilation (MV) may also cause lung damage which can lead to or worsen bronchopulmonary dysplasia (BPD) [4]. If in the past the “traditional” method of surfactant administration through the endotracheal tube (ETT) by intubation followed by MV was used exclusively, now the INSURE technique (INtubation-SURfactant administration-Extubation) is widely promoted, with current trends opting to avoid intubation.

It is currently considered that supporting transition rather than resuscitation, using a less invasive exogenous surfactant administration protocol (LISA), avoiding MV as much as possible to minimize mechanical lung damage and early initiation of nasal continuous positive airway pressure (nCPAP), may reduce mortality and/or the risk of early complications (e.g., air leaks, intraventricular hemorrhage) and/or late complications (e.g., BPD) [5,6].

To avoid the negative effects of intubation and MV, less invasive procedures of surfactant replacement with thin catheters (feeding tubes or vascular catheters) have been in use in our unit in the last 7 years, in addition to the traditional and INSURE procedures. Therefore, the aim of our study was to quantify our experience, to compare different strategies of surfactant administration and to investigate which method of surfactant administration is associated with less morbidity.

The primary outcome was the need for endotracheal intubation and mechanical ventilation within seventy-two hours after procedure. The secondary outcomes were complications of RDS and death before discharge.

## 2. Materials and Methods

### 2.1. Study Design and Patients

Over the study period, a total of 135 preterm infants with early rescue surfactant administration (in the first 2 h of life) for RDS were eligible for inclusion in the study. The infants were treated in an academic, Level III (regional) Neonatal Intensive Care Unit (NICU), over a 3-year period between 1 December 2018 and 1 December 2021. The study was approved by the ethics committee of County Emergency Hospital of Targu Mures (35517/11.2018). Written informed consent was obtained from parents.

The inclusion criteria in the study were: infants born in less than 32 weeks of gestational age (GA) (22/(0/7), to 31/(6/7) weeks of GA, with an estimation made on the bases of the modified Ballard score [7]) for the early rescue surfactant administration for RDS. We enrolled only the infants who were born in our neonatology department.

Exclusion criteria from the study were: preterm infants born in less than 32 weeks of GA with congenital malformations (cyanotic heart disease, diaphragmatic hernia), chromosomal abnormalities or congenital infection, infants who received surfactant after two hours of life and infants born outside of our unit.

**Figure 1:**
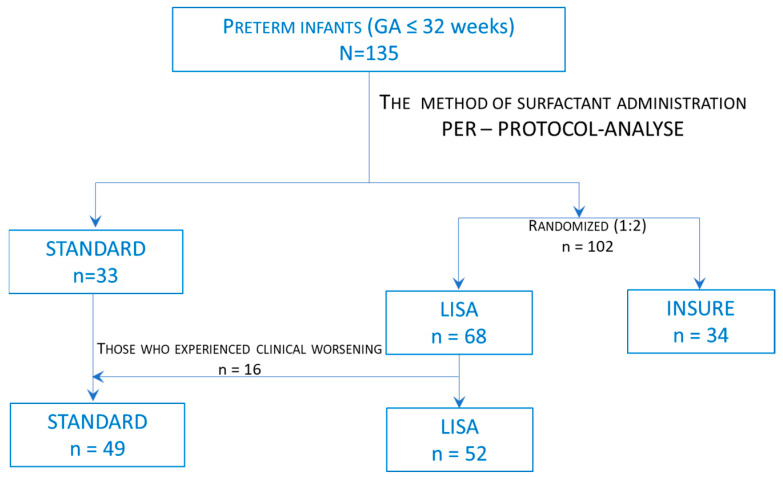
Flow chart for study groups.

The preterm infants were divided into three study groups according to the method of surfactant administration. The method of surfactant administration was set up according to the department’s protocols. Thus, 33 infants received the surfactant by the standard method and 102 infants should have received the surfactant by the INSURE and LISA methods. The selection of patients for the INSURE or LISA methods was decided by randomization (1:2 ratio; 34 vs. 68). The patient randomization was performed at birth. We used the sealed envelopes method and randomization was performed by an independent blinded study nurse. In turn, 16 infants with spontaneous breathing at birth who should have received surfactant by the LISA method—subsequently, in the first two hours of life—presented clinical worsening (severe apnea with cyanosis, shallow breathing/severe work of breathing, hemodynamic instability) (Figure 1). Thus, they needed intubation and received surfactant by the standard method. Finally, 49 infants received surfactant by the standard method (standard group), 34 infants received surfactant by the INSURE method (INSURE group), and 52 infants received surfactant by the LISA method (LISA group) (Figure 1).

### 2.2. Study Intervention

Along with the strict application of a resuscitation algorithm for preterm infants [2], depending on their condition at birth, spontaneously breathing infants were given a minimum 5 min alveolar recruitment with a T-piece resuscitator (Neopuff infant resuscitator; Fisher & Paykel Healthcare, Inc., Auckland, New Zealand). Positive end expiratory pressure (PEEP) was set at 5 cmH_2_O.

An amount of 100 to 200 mg/kg surfactant (poractant alpha, Curosurf, Chiesi Pharmaceuticals, Parma, Italy) was used in all patients. Intravenous Caffeine citrate (Peyona, Chiesi Farmaceutici SpA) was administered with a loading dose of 20 mg/kg before the INSURE and LISA procedures and continued with a maintenance dose of 5 mg/kg per day.

Patients without spontaneous breathing at birth and/or bradycardia who needed intubation in the delivery room for resuscitation or those with severe respiratory distress with desaturations below target saturation for a given postnatal age detected by pulse oximetry received surfactant by the standard method through the endotracheal tube (ETT) at doses of at least 100 mg/kg (100–200 mg/kg), followed by MV. The ETT position was evaluated by auscultation of the chest and/or X-ray. Mechanical ventilation was performed following the department’s protocol, with weaning from MV as soon as possible (FiO_2_ of less than 0.3 and mean airway pressure of less than 10 cm H_2_O).

Spontaneously breathing preterm infants with RDS received surfactant via either the INSURE or LISA method. 

INSURE was defined as transient intubation for surfactant administration through the ETT without sedatives and premedication, followed by immediate extubation after 1–2 min of positive pressure ventilation with a self-inflating bag, followed by nCPAP/noninvasive intermittent positive pressure ventilation (NIPPV). The surfactant was given in a dose of 200 mg/Kg within 1–3 min in small boluses under the continuous monitoring of heart rate and saturations by pulse oximetry. INSURE failure was defined as the need for MV within seventy-two hours after surfactant treatment. The criteria for intubation and initiating MV post-INSURE were the requirements of FiO_2_ > 0.4 to maintain O_2_ saturation above 90% or persistent apnea and/or increased respiratory distress.

LISA was defined as surfactant administration while the infant is breathing on nCPAP, using a 5–6 Fr. feeding tube to pass into the trachea through the vocal cords 1–2 cm under direct laryngoscopy with no sedatives, no premedication and without using a Magill forceps. The surfactant was given in a dose of 200 mg/Kg within 1–3 min in small boluses, under the continuous monitoring of heart rates and saturations by pulse oximetry, the feeding tube being removed at once after the procedure. If catheterization would have been not possible within 30 s, the procedure would have been stopped and attempted once again. LISA was followed by nCPAP/NIPPV. LISA failure was defined as need for MV within seventy-two hours after surfactant treatment. The criteria of intubation and MV were the same as in INSURE group.

RDS is usually defined as respiratory distress appearing within the first 24 h of life, with complete, sustained, and prompt response to surfactant, lung recruitment, or both (additional non-mandatory criteria are lung imaging supporting the diagnosis, lamellar body counts ≤ 30,000/mm^3^, or both) [8]. RDS was diagnosed based on clinical criteria (dyspnea, tachypnea, progressive grunting, retractions and cyanosis), supplemental oxygen requirements (fraction of inspired oxygen (FiO_2_) more than 0.30 to maintain O_2_ saturation in the range of 88–94%), [5,9] radiologic evidence of RDS in chest X-rays, and/or altered blood gases tests (respiratory acidosis with pH less than 7.2 and PCO_2_ more than 65 mmHg). The severity (mild/moderate/severe) of RDS was defined according to the clinical classification and the Silverman Scoring System (score 0–3: mild; score 4–6: moderate; and score < 6: severe respiratory distress).

Bronchopulmonary dysplasia (BPD) was defined as oxygen requirement at 36 weeks post-menstrual age [8,9,10].

Necrotizing enterocolitis (NEC) was defined by using the Bell classification [11].

Intraventricular hemorrhage (IVH) was defined and graded according to Papile et al. [12] Cranial ultrasounds were performed on day 1, 2, 3, 4, 7, 14 and 21 for all patients.

The diagnosis of patent ductus arteriosus (PDA) was based on the clinical and echocardiographic parameters [10,11,12,13].

Retinopathy of prematurity (ROP) was defined using the standard classification [14].

Neonatal sepsis was defined as the presence of clinical signs and symptoms of sepsis with the isolation of pathogens from blood, cerebrospinal fluid, and urine from birth to 28 days of life. Probable sepsis was defined as the presence of the clinical picture of sepsis, without growth of any pathogen from blood, cerebrospinal fluid, or urine but with modified inflammatory and hematological markers.

Mortality was defined as death before discharge.

Infant Flow SiPAP (Viasys Healthcare, Palm Springs, CA, USA) and Medin (Medical Innovations GmbH, Lindbergh Strasse, Puchheim, Germany) with binasal prongs were used to provide nCPAP. PEEP levels ranged between 5 and 8 cm H_2_O and FiO_2_ was adjusted to keep oxygen saturation of 90–95%.

Leoni plus (Löwenstein Medical GmbH & Co. KG, Arzbacher,56130 Bad Ems, Germany) and Babylog 8000 plus (Drager, Germany) ventilators were used for MV. The rescue MV modes for post-INSURE or post-LISA respiratory failure were time cycled pressure limited conventional ventilation (Synchronized intermittent mandatory ventilation (SIMV) ± Pressure support (PS)) and high frequency oscillatory ventilation (HFOV).

### 2.3. Statistical Analysis

The study groups were analyzed according to per-protocol analysis for the surfactant administration method. The statistical analysis of the variables of interest was conducted in SPSS v.29 (IBM Ireland Product Distribution Limited, IBM House, Shelbourne Road, Ballsbridge, Dublin 4, Ireland). For continuous variables, we assessed the averages and standard deviation (mean ± SD) or the median and interquartile range (IQR) depending on the normal distribution of the values. The comparisons between the statistical groups were performed with the Kruskal–Wallis test for continuous variables or Student’s *t*-test. The Levene test was used to assess the homogeneity of variances. For qualitative variables, we analyzed frequencies (absolute and relative %) and performed comparisons between groups based on the results of non-parametric tests (Pearson Chi-square). The association of the surfactant administration method with the clinical course of preterm infants was assessed based on the odd ratio (OR) resulting from a univariate logistic regression. The Kaplan–Meier method was used to evaluate overall survival (OS), and the Log-Rank test was used to make comparisons. The threshold for statistical significance was set at *p* < 0.05.

## 3. Results

In the three groups of infants there were no significant differences in the mother’s age (*p* = 0.920), the distribution according to the gender of the infants (*p* = 0.6112), the type of delivery (*p* = 0.9821) and the antenatal corticosteroids administration (*p* = 0.1261). The average GA of the infants who received the surfactant by the standard method was significantly (*p* < 0.001) lower (25.8 w ± 2.5) compared to the GA of the infants who were given the surfactant by LISA (27.6 w ± 2) or INSURE (28.1 w ± 1.9) (Table 1).

The average birth weight (BW) of infants who were given the surfactant by the standard method was 828.4 g ± 325.5, with a minimum of 450 g and a maximum of 2150 g. Infants who received the surfactant by LISA (1043.8 g ± 335.9) and INSURE (1095.3 g ± 272.4) had significantly higher BW (*p* = 0.0002) compared to the BW of those who were given the surfactant by the standard method (Table 1). The standard group also presented with lower Apgar scores and a higher need of resuscitation.

Significant desaturation, surfactant reflux, bradycardia and apnea effects during the procedure were observed in 21.15% of infants who received the surfactant by the LISA method and in 20.54% of infants from the INSURE group. 

The duration of MV needed after the procedure was significantly shorter (Student’s *t*-test Test, *p* = 0.0103) in the case of the LISA group (89.2 h ± 33.9) compared to the INSURE group (143.8 h ± 54.5).

The method of surfactant administration was significantly associated with the presence of major neonatal morbidities (*p* = 0.0168). In the LISA group, 61.5% of these infants had morbidities and in the INSURE group they had 76.5% (Table 1). 

The analysis of morbidities did not show significant differences amongst groups in frequencies of cases of BPD (*p* = 0.2541), NEC (*p* = 0.7218) and PDA (*p* = 0.8254) (Table 1).

In the analyzed group, 87.8% of these infants had morbidities (Table 2). The analysis of the morbidities found in infants who were given surfactant by the standard method showed a higher incidence of pneumothorax (18.4%), IVH (55.1%), IVH grade 3 and 4 (26.5%), ventriculomegaly (24.5%), sepsis/probable sepsis (23.4%) and ROP (56.7%). The length of stay in the NICU was higher in the standard group (Table 2).

The severity (mild/moderate/severe) of RDS in premature infants in the analyzed groups was evaluated clinically according to the Silverman scoring system (score 0–3: mild; score 4–6: moderate; and score < 6: severe respiratory distress) (Table 3). An amount of 25% of infants from the LISA group needed MV (failure of LISA), while 38.2% of those receiving surfactant by the INSURE method required re-intubation and MV (failure of INSURE) (Table 3).

### 3.1. Association of Surfactant Administration Methods and Gestational Age with the Subsequent Need for Mechanical Ventilation and the Occurrence of Major Neonatal Morbidities

We analyzed the association between GA, the need for subsequent MV and the major neonatal morbidities depending on the method of surfactant administration (Table 4). Thus, for the LISA method the analysis showed a significant association between the need for MV (failure of LISA) within 72 h after the procedure and GA (*p* = 0.0133). Within the same group, the need for MV was significantly more common for infants with a GA of less than 25 weeks (80%) and between 25 and 29 weeks (22.2%).

In the case of surfactant administration by the INSURE method, the frequency of cases that needed MV within 72 h after procedure did not show significant differences depending on GA (*p* = 0.9824). Thus, we can appreciate that failure of the INSURE method is not significantly associated with the infant’s GA (Table 4).

Re-administration of the surfactant was significantly associated (*p* < 0.01) with a GA over 26 weeks regardless of the method of administration. Therefore, it was significantly more common to repeat the surfactant dose in infants with a GA of 30–32 weeks included from the standard group (60%). Regarding the LISA method, a second dose of the surfactant was needed in 11.1% of infants with a GA between 26 and 29 weeks. In the INSURE group, it was necessary to repeat the surfactant dose for approximately 20% of the infants with a GA between 26 and 32 weeks (Table 4).

The presence of major neonatal morbidities was significantly associated with low a GA (22–25 weeks) in the case of both LISA (80%) and INSURE (100%). It was noted that, in the standard group, the frequency of morbidities had significantly increased regardless of GA. The occurrence of pneumothorax was significantly higher (*p* = 0.0364) in infants with a GA of 30–32 weeks with surfactant administration by the standard method (40%). The frequency of pneumothorax occurrence was lower in the LISA group (5.6%, GA: 26–29 weeks), while 20.5% of the infants in the INSURE group with a GA over 26 weeks experienced a pneumothorax. BPD had a high frequency in infants who received the surfactant by the standard method, regardless of their GA. In both the LISA (40%) and INSURE (80%) groups, BPD was significantly more common in infants with a GA of 22–26 weeks (Table 4).

The frequency of cases of severe IVH was significantly increased (*p* = 0.0284) for a GA between 22 and 29 weeks. It is noted that IVH grade 3 and 4 was present in the LISA group only in infants with a low GA (22–25 weeks), while in the INSURE group there was a small number (insignificant) of cases with GA’s between 26 and 32 weeks (Table 4).

The presence of sepsis/probable sepsis was significantly associated with the low GA of infants in the INSURE group (*p* = 0.0151) and the standard group (*p* = 0.0028), but no significant association with the GA was found in the case of LISA (*p =* 0.8305) (Table 4).

The outcome of the preterm infants was not significantly associated with GA in the INSURE group (*p* = 0.1566). In LISA group (40%—GA: 22–25 weeks) and standard group (62.9%—GA: 22–25 weeks), the frequency of deaths was significantly higher in very preterm infants (Table 4).

### 3.2. Association of the Surfactant Administration Method (LISA and INSURE) and Gestational Age, with the Clinical Evolution of Infants: Need of MV, Need for Subsequent Surfactant Doses, Morbidities, Sepsis/Proven Sepsis and Deaths

The effectiveness of LISA in the avoidance MV within seventy-two hours of life was strongly dependent on GA. Consequently, a GA of less than 26 weeks significantly increases the need for MV (OR = 2.74, *p* < 0.001), while a GA over 26 weeks significantly increased the need of repeating the surfactant dose (Table 5).

The LISA method significantly reduces the need for subsequent doses of surfactant compared to the INSURE method (OR = 0.538, *p* = 0.019). The analysis also showed that the greater a GA of 26 weeks the greater the need for the repeated administration of surfactant (OR = 1.134, *p* = 0.021) (Table 5).

The occurrence of major neonatal morbidities in preterm infants requiring surfactant administration was significantly lower in the LISA group compared to the INSURE group (OR = 0.492, *p* = 0.015). The GA of less than 26 wks. increased the frequency of morbidities in this vulnerable category of infants (OR = 2.821, *p* = 0.026) (Table 5).

Analyzing a possible association with the occurrence of sepsis/probable sepsis, a GA of less than 26 weeks was identified as a significant risk factor (OR = 3.065, *p* = 0.003). The LISA group significantly decreased the chance of sepsis/probable sepsis compared to the INSURE method (OR = 0.609, *p* = 0.042) (Table 5).

Regarding the number of deaths, a significant association with a GA of less than 26 weeks was noted (OR = 5.367, *p* < 0.001). The results indicated a significant decrease in the chance of death of infants treated by the LISA method compared to those treated by the INSURE method (OR = 0.640, *p* = 0.036) (Table 5).

**Figure 2:**
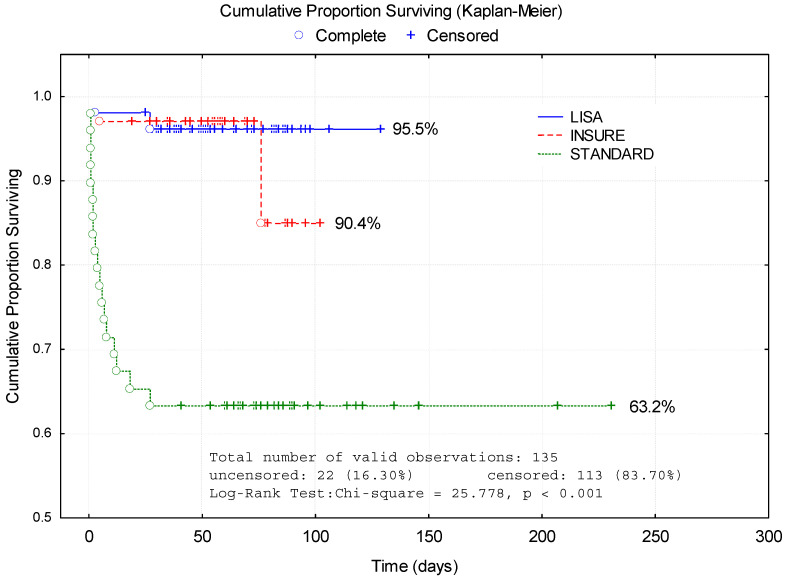
Comparative analysis of Kaplan–Meier curves between methods of surfactant administration.

The survival rate was significantly (*p* < 0.01) higher in the LISA group (95.5%) compared to the INSURE group (90.4%) (Figure 2).

## 4. Discussion

The significant achievements in neonatal intensive care over the past decades are highlighted by improvements in the survival rate of very preterm infants. The major goal for neonatologists has become to limit the incidence and severity of complications of prematurity (e.g., RDS, BPD, IVH, PVL, NEC and ROP), especially the disabling ones that could impact overall quality of life [14,15]. By far, RDS is one of the major causes of neonatal mortality and morbidity in preterm infants [16,17]. For a long time, the standard approach of RDS was surfactant therapy during intermittent positive pressure ventilation (IPPV). Thus, administration of surfactant became one of the most common procedures performed in very preterm infants. As MV has been shown to be a risk factor for BPD due to airway and lung injury (barotrauma/volumtrauma/atelectrauma) produced, even in the case of a brief period of ventilation, different ventilation strategies have been developed to address this issue over time [18,19,20,21,22]. In order to decrease the need for intubation and MV, various noninvasive ventilation strategies have been widely introduced (e.g., CPAP, SIPPV and high-flow nasal cannula) [23,24,25,26,27,28]. Looking at the current recommendations of the European Consensus Guidelines on the Management of Respiratory Distress Syndrome—2019 Update, CPAP has been widely recommended to stabilize preterm infants in the delivery room and as a respiratory support before and after surfactant replacement. Hence, nCPAP is slowly replacing the MV and becoming the new standard of care. Current recommendations are to administer surfactant as early rescue therapy in the first two hours after birth, but only if respiratory distress is present [3,29]. There are still controversies about the optimal method of surfactant administration. Although the INSURE method has been routinely used since the 1990s, it involves short-term endotracheal intubation followed by a brief period of bag and mask ventilation or MV. Additionally, the iatrogenic risks of intubation are well known and, consequently, this widespread method of surfactant administration was gradually replaced by various techniques that avoided intubation per se. The terminology of the techniques differs (e.g., LISA—less invasive surfactant administration; MIST—minimally invasive surfactant therapy), but the principle is the same, respectively, the replacement of ETT with a smaller dimension device (e.g., feeding tube, umbilical catheter, Angio catheter, LISA Cath, Neo fact, Surf Cath). It is important to mention that all these methods can be utilized only in infants presenting with spontaneous breathing. There is still controversy in the literature regarding the type of catheter and the necessity of use of the Magill guide forceps or premedication (Atropine—to reduce oropharyngeal secretions and vagal reflex, analgesia with Fentanyl). Additionally, there are several methods of surfactant administration using feeding tubes (Cologne method, Take Care method, SONSURE method) or 16 g Angio catheters (Hobart method), which have been described. Noninvasive surfactant administration has been the subject of many studies in recent years (e.g., LISA vs. INSURE, INSURE vs. the standard method, LISA vs. the standard method) [30,31,32,33,34,35]. In our country, our NICU was the first to use the LISA method of surfactant replacement as a first-line technique. Our method of surfactant administration differs from other methods described by using low-cost devices available in all facilities and totally avoiding sedation, premedication and supplemental forceps, thus, minimizing invasivity.

In the present study, we compared three different strategies of surfactant administration: the standard method via endotracheal intubation, followed by MV and two methods of delivering surfactant in a gentler way with LISA (with feeding tube) and INSURE, followed by non-invasive ventilation. Regarding the standard group, the average GA and BW of the infants who received the surfactant was significantly (*p* < 0.00; *p* = 0.0002, respectively) lower compared to the GA and BW of infants who were given surfactant by LISA or INSURE. The standard method was also associated with significantly lower Apgar scores (*p* < 0.001), a higher need of resuscitation, repeated surfactant doses and longer mean times of MV, probably due to lower a GA that needed more support in all.

Regarding the primary outcome of the present study, the requirement for endotracheal intubation and MV within seventy-two hours after the procedure was significantly associated with the method of the surfactant administration. Comparing LISA with INSURE, we found that the need for MV was significantly lower in the LISA group (25% vs. 38.2%, respectively), while the mean times of MV was significantly shorter in the LISA group (89.2 h ± 33.9 vs. 143.8 h ± 54.5, respectively) as compared with the INSURE group. LISA group infants had a lesser need for MV (OR = 0.538; 95% CI: 0.212 to 0.730, *p* = 0.019) and repeated doses of surfactant (OR = 0.389; 95% CI: 0.101 to 0.894, *p* = 0.017) than the INSURE group infants. Previous reports have shown similar results. A study conducted by Göpel et al. included 1103 infants born under 32 weeks of gestation and treated with LISA at 37 centers in Germany, showing that LISA was associated with lower rates of MV (41% versus 62%, *p* < 0.001) [36]. Krajewski et al. in a study involving 26 preterm infants with RDS receiving surfactant via a thin catheter reported a significant reduction in the need for MV (19.2% vs. 65%, *p* < 0.05) compared to the INSURE technique [37]. Two meta-analyses also showed a significant reduction in the need for MV within the first 72 h, and a reduction in the duration of MV in the LISA group compared with INSURE [6,38]. On the contrary, Aguar et al. in a study of 44 preterm infants with RDS receiving surfactant via a gastric tube reported no significant difference in the need for mechanical MV (25% vs. 33%, *p* = 0.44) and the duration of required MV (115 h vs. 150 h, *p* > 0.05) between the LISA and INSURE methods of delivering surfactant [31].

Regarding the secondary outcomes of the present study, we found less morbidities (OR = 0.492; 95% CI: 0.187 to 0.892; *p* = 0.015) and deaths before discharge (OR = 0.640; 95% CI: 0.386 to 0.774; *p* = 0.035) in the LISA group compared with the INSURE group. We also found a significant association of the standard method of surfactant administration with major neonatal morbidities (Pneumothorax, IVH, severe IVH, PVL, ventriculomegaly, ROP, sepsis/probable sepsis), deaths and the length of stay in neonatal intensive care. The analysis of morbidities found in infants who were given surfactant by the LISA method compared with the INSURE method showed lower incidence of Pneumothorax (3.9% vs. 8.8%), IVH (17.3% vs. 23.5%), IVH grade 3 and 4 (3.9% vs. 5.9%), ventriculomegaly (0% vs. 8.8%), sepsis/probable sepsis (11.5% vs. 17.7%), ROP (16.7% vs. 26.7%) and deaths (3.9% vs. 5.9%). There were no significant differences between groups in the frequencies of cases of BPD, NEC, PDA and length of stay in NICU. Kribs [39] compared the outcomes of the LISA method applied in infants born under 31 weeks of gestation with those of standard surfactant therapy in the same category of infants. The results showed the LISA group had a significant reduction in the need for MV within the first 72 h (29% vs. 53%, respectively; *p* < 0.001) and a decrease in BPD incidence (10.9% vs. 17.5%, respectively; *p* = 0.004). Another study involving LISA via thin catheter vs. INSURE proved significant reductions in BPD (15.4% vs. 40%, *p* < 0.05) and a lower incidence of other complications of prematurity, such as NEC (11.5% vs. 23.3%) and ROP (3.9% vs. 11.7%) [37]. LISA was associated with a reduced risk for adverse outcomes such as BPD (OR 0.55; 95% CI: 0.49–0.62, *p*  <  0.001), IVH grade II-IV (OR 0.55; 95% CI: 0.48–0.64, *p* < 0.001), ROP (OR 0.62; 95% CI: 0.45–0.85, *p*  <  0.001) and mortality (OR 0.66; 95% CI: 0.51–0.84, *p*  <  0.001) in a study conducted by Härtel et al. [40]. One of the main limitations of the study was the small number of cases included, which were studied according to per-protocol analysis.

## 5. Conclusions

This study highlights that the INSURE and LISA methods do not significantly decrease the number of BPD cases, but the latter has been shown to be overall more efficient than INSURE and reduces the need of MV and the severe morbidities in premature infants with RDS. Our method of surfactant administration differs from other methods described by the use of low-cost devices available in all facilities and the total avoidance of sedation, premedication and supplemental forceps, thus, minimizing invasivity.

Concerning the best choice for surfactant administration under debate, our study underlines once more that LISA has no more complications than INSURE, has the benefit of minimal invasivity in surfactant replacement and has potential in improving preterm infant outcomes. LISA requires certain skills and experience to be safely administered, but it is a method that shows promising results in the treatment of RDS. LISA can be used until a totally non-invasive method of surfactant administering (possibly by nebulization) is developed.

## Figures and Tables

**Table 1:** Comparison of clinical characteristics between the groups depending on the method of administration of the surfactant.

Baseline Characteristics	Study Group (n = 135)			*p*-Value
	LISA (n = 52)	INSURE (n = 34)	STANDARD (n = 49)	
Mother’s age: years (mean ± SD) ^†^	30.2 ± 5.8	29.7 ± 7.1	29.7 ± 6	0.920
Gender (F/M), n (%) ^‡^	27/25	15/19	23/26	0.6112
	(51.9/48.1)	(44.1/55.9)	(46.9/53.1)	
GA, weeks †	27.6 ± 2	28.1 ± 1.9	25.8 ± 2.5	< 0.001 *
22–25 weeks	5 (9.6)	5 (14.7)	27 (55.1)	
26–29 weeks	36 (69.2)	19 (55.9)	17 (34.7)	
30–32 weeks	11 (21.2)	10 (24.4)	5 (10.2)	
BW, g, (mean ± SD) ^†^	1043.8 ± 335.9	1095.3 ± 272.4	828.4 ± 325.5	0.0002 *
LGA/AGA/SGA, n (%) ^‡^	1/44/7	0/32/2	1/47/1	0.1693
	(1.9/84.6/13.5)	(0/94.1/5.9)	(2.1/959/2.1)	
Cesarian section/vaginal delivery, n (%)	32/20	21/13	31/18	0.9821
	(61.5/38.5)	(61.8/38.2)	(63.3/36.7)	
Apgar score, median (IQR)				
1 min	7 (6–7)	6 (5–7)	3 (2–5)	<0.001 *
5 min	8 (8–8)	8 (7–8)	5 (5–7)	0.0124 *
**Clinical Characteristics**				
Duration of MV, hours (mean ± SD) ^#^	89.2 ± 33.9	143.8 ± 54.5		0.0103 *
Antenatal corticosteroids, n (%) ^‡^	36 (69.2)	17 (50)		0.2214
Need for subsequent surfactant doses, n (%) ^‡^	4 (7.7)	6 (17.7)		0.0219 *
Morbidity, n (%) ^‡^	32 (61.5)	26 (76.5)		0.0168 *
Pneumothorax, n (%) ^‡^	2 (3.9)	3 (8.8)		0.0351 *
BPD, n (%) ^‡^	13 (25)	13 (38.2)		0.2541
NEC, n (%) ^‡^	6 (11.5)	5 (14.7)		0.7218
IVH, n (%) ^‡^	9 (17.3)	8 (23.5)		0.0054 *
IVH 3–4, n (%) ^‡^	2 (3.9)	2 (5.9)		0.0038 *
PVL, n (%) ^‡^	0 (0)	4 (11.8)		0.0269 *
PDA, n (%) ^‡^	14 (26.9)	11 (32.4)		0.8254
Ventriculomegaly, n (%) ^‡^	0 (0)	3 (8.8)		0.0051 *
Sepsis/probable sepsis, n (%) ^‡^	6 (11.5)	6 (17.7)		0.0356 *
ROP, n (%) ^‡^	5 (16.7)	8 (26.7)		0.0397 *
NICU, days, median (IQR) ^#^	19 (14–25)	20 (14–27)		0.0242 *
Deaths, n (%) ^‡^	2 (3.9)	2 (5.9)		0.0059 *

LISA: Less Invasive Surfactant Administration; INSURE: INtubation-SURfactant administration-Extubation; Continuous variables were expressed as: mean ± standard deviation or median (IQR); categorical variables: number (%); n: number; GA: gestational age; M: male; F: female; BW: birth weight; LGA: large for gestational age; AGA: appropriate for gestational age; SGA: small for gestational age; MV: mechanical ventilation; BPD: bronchopulmonary dysplasia; NEC: necrotizing enterocolitis; IVH: intraventricular hemorrhage; PVL: periventricular leukomalacia; PDA: patent ductus arteriosus; ROP: retinopathy of prematurity; NICU: Neonatal Intensive Care Unit; IQR: interquartile range; † Kruskal–Wallis Test for continuous variables. # Student’s *t*-test. ‡ Pearson Chi-square test; * Marked effects are significant at *p* < 0.05.

**Table 2:** Clinical characteristics of premature infants who were administered surfactant by the standard method.

	STANDARD, (n = 49)
Duration of MV, hours (mean ± SD)	217.6 ± 173
Antenatal corticosteroids, n (%)	26 (53.1)
Need for subsequent surfactant doses, n (%)	12 (24.5)
Morbidity, n (%)	43 (87.8)
Pneumothorax, n (%)	9 (18.4)
BPD, n (%)	22 (44.9)
NEC, n (%)	9 (18.4)
IVH, n (%)	27 (55.1)
IVH 3–4, n (%)	13 (26.5)
PVL, n (%)	3 (6.1)
PDA, n (%)	12 (24.5)
Ventriculomegaly, n (%)	12 (24.5)
Sepsis/probable sepsis, n (%)	11 (23.4)
ROP, n (%)	17 (56.7)
NICU, days, median (IQR)	26 (7–43)
Deaths, n (%)	18 (36.7)

Continuous variables were expressed as: mean ± standard deviation; categorical variables: number (%); n: number; MV: mechanical ventilation; BPD: bronchopulmonary dysplasia; NEC: necrotizing enterocolitis; IVH: intraventricular hemorrhage; PVL: periventricular leukomalacia; PDA: patent ductus arteriosus; ROP: retinopathy of prematurity; NICU: Neonatal Intensive Care Unit; IQR: interquartile range.

**Table 3:** Respiratory distress syndrome and need of MV depending on the method of administration of the surfactant.

	Study Group (n = 135)		
	LISA (n = 52)	INSURE (n = 34)	STANDARD (n = 49)
RDS, n (%)	8/39/5	0/27/7	1/31/17
mild/moderate/severe	(15.4/75/9.6)	(0/79.4/20.6)	(2.1/63.3/34.6)
Need of MV (<72 h), n (%)	13 (25)	13 (38.2)	49 (100)

n: Number; MV: Mechanical ventilation.

**Table 4:** Association of surfactant administration methods and gestational age with the subsequent need for mechanical ventilation and the occurrence of major neonatal morbidities.

	GA, Weeks			*p*-Value ^‡^
	22–25 Weeks	26–29 Weeks	30–32 Weeks	
**Need of MV (<72 h), n (%)**				
LISA, n _MV_/n _total_	4/5 (80)	8/36 (22.2)	1/11 (9.1)	0.0133 *
INSURE, n _MV_/n _total_	2/5 (40)	7/19 (36.8)	4/10 (40)	0.9824
**Need for subsequent surfactant doses**				
LISA, n _subsequent doses_/n _total_	0/5 (0)	4/36 (11.1)	0/11 (0)	0.0025 *
INSURE, n _subsequent doses_/n _total_	0/5 (0)	4/19 (21.1)	2/10 (20)	0.0017 *
STANDARD, n _subsequent doses_/n _total_	5/27 (18.5)	4/17 (23.5)	3/5 (60)	0.0108 *
**Morbidity, n (%)**				
LISA, n _morbidity/_n _total_	4/5 (80)	22/36 (61.1)	6/11 (54.6)	0.0174 *
INSURE, n _morbidity/_n _total_	5/5 (100)	14/19 (73.7)	7/10 (70)	0.0351 *
STANDARD, n _morbidity/_n _total_	23/27 (85.2)	15/17 (88.2)	5/5 (100)	0.4802
**Pneumothorax, n (%)**				
LISA, n _Pneumothorax_/n _total_	0/5 (0)	2/36 (5.6)	0/11 (0)	0.4709
INSURE, n _Pneumothorax/_n _total_	0/5 (0)	2/19 (10.5)	1/10 (10)	0.6049
STANDARD, n _Pneumothorax/_n _total_	4/27 (14.8)	3/17 (17.7)	2/5 (40)	0.0364 *
**BPD, n (%)**				
LISA, n _BPD_/n _total_	2/5 (40)	10/36 (27.8)	1/11 (9.1)	0.0031 *
INSURE, n _BPD_/n _total_	4/5 (80)	8/19 (42.1)	1/10 (10)	0.0196 *
STANDARD, n _BPD_/n _total_	11/27 (40.7)	8/17 (47.1)	3/5 (60)	0.7116
**IVH grade 3–4, n (%)**				
LISA, n _IVH_/n _total_	2/5 (40)	0/36 (0)	0/11 (0)	0.0060 *
INSURE, n _IVH_/n _total_	0/5 (0)	1/19 (5.3)	1/10 (10)	0.6453
STANDARD, n _IVH_/n _total_	9/27 (33.3)	4/17 (23.5)	0/5 (0)	0.0284 *
**PVL, n (%)**				
LISA, n _PVL_/n _total_	-	-	-	-
INSURE, n _PVL_/n _total_	0/5 (0)	3/19 (15.8)	1/10 (10)	0.4596
STANDARD, n _PVL_/n _total_	1/27 (3.7)	1/17 (5.9)	1/5 (20)	0.4948
**PDA, n (%)**				
LISA, n _PDA_/n _total_	2/5 (40)	9/36 (25)	3/11 (27.3)	0.7905
INSURE, n _PDA_/n _total_	2/5 (40)	4/19 (21.1)	5/10 (50)	0.2649
STANDARD, n _PDA_/n _total_	3/27 (11.1)	8/17 (47.1)	1/5 (20)	0.0272 *
**Sepsis/probable sepsis, n (%)**				
LISA, n_sepsis/probable sepsis_/n _total_	1/5 (10)	4/36 (11.1)	1/11 (9.1)	0.8305
INSURE, n_sepsis/probable sepsis_/n _total_	3/5 (60)	3/19 (15.8)	0/10 (0)	0.0151 *
STANDARD, n_sepsis/probable sepsis_/n _total_	5/27 (18.5)	6/16 (37.5)	0/4 (0)	0.0028 *
**Deaths, n (%)**				
LISA, n _Deaths/_n _total_	2/5 (40)	0/36 (0)	0/11 (0)	0.0047 *
INSURE, n _Deaths/_n _total_	1/5 (20)	0/19 (0)	1/10 (10)	0.1566
STANDARD, n _Deaths/_n _total_	17/27 (62.9)	1/17 (5.9)	0/5 (0)	0.00002 *
**ROP, n (%)**				
LISA, n _ROP/_n _total_	2/5 (40)	6/36 (16.7)	0/11 (0)	0.0409 *
INSURE, n _ROP/_n _total_	1/5 (20)	2/19 (105)	2/10 (20)	0.7422
STANDARD, n _ROP/_n _total_	8/27 (29.6)	8/17 (47.1)	1/5 (20)	0.3811
**NICU, days**				
LISA, median (IQR)	27 (23–27)	21 (15–27)	12 (10–20)	0.0329 *
INSURE, median (IQR)	25 (22–27)	21 (14–26)	17 (13–19)	0.0414 *
STANDARD, median (IQR)	11 (2–28)	40 (26–44)	29 (28–60)	0.0103 *

LISA: Less Invasive Surfactant Administration; INSURE: INtubation-SURfactant administration-Extubation; n: number; GA: gestational age; MV: mechanical ventilation; BPD: bronchopulmonary dysplasia; IVH: intraventricular hemorrhage; PVL: periventricular leukomalacia; PDA: patent ductus arteriosus; NICU: Neonatal Intensive Care Unit; IQR: interquartile range; ‡ Pearson Chi-square test; * Marked effects are significant at *p* < 0.05.

**Table 5:** Univariate logistic regression for prediction.

Univariate Analysis (n _LISA_ = 52; n _INSURE_ = 34)	Odds Ratio (95% Confidence Interval)	SE	*p*-Value
**Need of MV** (<72 h)			
LISA (ref. INSURE)	0.538 (0.212–0.730)	0.047	0.019 *
GA, weeks (ref. GA > 26 weeks)	2.735 (1.608–5.971)	0.183	<0.001 *
**Need for subsequent surfactant doses**			
LISA (ref. INSURE)	0.389 (0.101–0.894)	0.088	0.017 *
GA, weeks (ref. GA < 26 weeks)	2.134 (1.391–5.380)	0.068	0.021 *
**Morbidity, n (%)**			
LISA (ref. INSURE)	0.492 (0.187–0.892)	0.049	0.015 *
GA, weeks (ref. GA > 26 weeks)	2.821 (1.691–4.976)	0.065	0.026 *
**Sepsis/probable sepsis, n (%)**			
LISA (ref. INSURE)	0.609 (0.179–0.988)	0.025	0.042 *
GA, weeks (ref. GA > 26 weeks)	3.065 (1.874–6.057)	0.034	0.003 *
**Deaths, n (%)**			
LISA (ref. INSURE)	0.640 (0.386–0.774)	0.025	0.036 *
GA, weeks (ref. GA > 26 weeks)	5.367 (3.245–7.551)	0.014	<0.001 *

LISA: Less Invasive Surfactant Administration; INSURE: INtubation-SURfactant administration-Extubation; GA: gestational age; * Marked effects are significant at *p* < 0.05.

## Data Availability

The data presented in this study are available on request from the corresponding authors.

## References

[1] Lopez E., Gascoin G., Flamant C., Merhi M., Tourneux P., Band O. (2013). Exogenous surfactant therapy in 2013: What is next? Who, when and how should we treat newborn infants in the future?. BMC Paediatr..

[2] Hatch L.D., Grubb P.H., Lea A.S., Walsh W.F., Markham M.H., Whitney G.M., Slaughter J.C., Stark A.R., Ely E.W. (2016). Endotracheal Intubation in Neonates: A Prospective Study of Adverse Safety Events in 162 Infants. J. Pediatr..

[3] Gomes Cordeiro A.M., Fernandes J.C., Troster E.J. (2004). Possible risk factors associated with moderate or severe airway injuries in children who underwent endotracheal intubation. Pediatr. Crit Care Med..

[4] Lademann H., Abshagen K., Janning A., Däbritz J., Olbertz D. (2021). Long-Term Outcome after Asphyxia and Therapeutic Hypothermia in Late Preterm Infants: A Pilot Study. Healthcare.

[5] Sweet D.G., Carnielli V., Greisen G., Hallman M., Ozek E., Te Pas A., Plavka R., Roehr C.C., Saugstad O.D., Simeoni U. (2019). European Consensus Guidelines on the Management of Respiratory Distress Syndrome—2019 Update. Neonatology.

[6] Aldana-Aguirre J.C., Pinto M., Featherstone R.M., Kumar M. (2017). Less invasive surfactant administration versus intubation for surfactant delivery in preterm infants with respiratory distress syndrome: A systematic review and meta-analysis. Arch. Dis. Child Fetal Neonatal Ed..

[7] Ballard J.L., Khoury J.C., Wedig K., Wang L., Eilers-Walsman B.L., Lipp R. (1991). New Ballard Score, expanded to include extremely premature infants. J. Pediatr..

[8] de Luca D., van Kaam A.H., Tingay D.G., Courtney S.E., Danhaive O., Carnielli V.P., Zimmermann L.J., Kneyber M.C.J., Tissieres P., Brierley J. (2017). The Montreux definition of neonatal ARDS: Biological and clinical background behind the description of a new entity. Lancet Respir. Med..

[9] Sola A., Golombek S.G., Montes Bueno M.T., Lemus-Varela L., Zuluaga C., Domínguez F., Baquero H., Young Sarmiento A.E., Natta D., Rodriguez Perez J.M. (2014). Safe oxygen saturation targeting and monitoring in preterm infants: Can we avoid hypoxia and hyperoxia?. Acta Paediatr..

[10] Jensen E.A., Dysart K., Gantz M.G. (2019). The diagnosis of bronchopulmonary dysplasia in very preterm infants: An evidence-based approach. Am. J. Respir. Crit Care Med..

[11] Bell M.J., Ternberg J.L., Feigin R.D., Keating J.P., Marshall R., Barton L., Brotherton T. (1978). Neonatal necrotizing enterocolitis. Therapeutic decisions based upon clinical staging. Ann. Surg..

[12] Papile L.A., Burstein J., Burstein R., Koffler H. (1978). Incidence and evolution of subependymal and intraventricular hemorrhage: A study of infants with birth weights less than 1500 gm. J. Pediatr..

[13] Gillam-Krakauer M., Reese J. (2018). Diagnosis and Management of Patent Ductus Arteriosus. Neoreviews.

[14] Ţarcă E., Roșu S.T., Cojocaru E., Trandafir L., Luca A.C., Rusu D., Ţarcă V. (2021). Socio-Epidemiological Factors with Negative Impact on Infant Morbidity, Mortality Rates, and the Occurrence of Birth Defects. Healthcare.

[15] Mavroudis I., Kazis D., Chowdhury R., Petridis F., Costa V., Balmus I.M., Ciobica A., Luca A.C., Radu I., Dobrin R.P. (2022). Post-Concussion Syndrome and Chronic Traumatic Encephalopathy: Narrative Review on the Neuropathology, Neuroimaging and Fluid Biomarkers. Diagnostics.

[16] International Committee for the Classification of Retinopathy of Prematurity (2005). The International Classification of Retinopathy of Prematurity revisited. Arch Ophthalmol..

[17] Verder H., Bohlin K., Kamper J., Lindwall R., Jonsson B. (2009). Nasal CPAP and surfactant for treatment of respiratory distress syndrome and prevention of bronchopulmonary dysplasia. Acta Paediatr..

[18] Cha J.H., Choi N., Kim J., Lee H.J., Na J.Y., Park H.K. (2022). Cystic Periventricular Leukomalacia Worsens Developmental Outcomes of Very-Low-Birth Weight Infants with Intraventricular Hemorrhage-A Nationwide Cohort Study. J. Clin. Med..

[19] Trembath A., Laughon M.M. (2012). Predictors of bronchopulmonary dysplasia. Clin Perinatol..

[20] Hunt K., Dassios T., Ali K., Greenough A. (2019). Volume targeting levels and work of breathing in infants with evolving or established bronchopulmonary dysplasia. Arch. Dis. Child Fetal Neonatal Ed..

[21] Escobar V., Soares D.S., Kreling J., Ferrari L.S., Felcar J.M., Camillo C.A., Probst V.S. (2020). Influence of time under mechanical ventilation on bronchopulmonary dysplasia severity in extremely preterm infants: A pilot study. BMC Pediatr..

[22] Choi Y.B., Lee J., Park J., Jun Y.H. (2018). Impact of prolonged mechanical ventilation in very low birth weight infants: Results from a National Cohort Study. J. Pediatr..

[23] Ho J.J., Subramaniam P., Henderson-Smart D.J., Davis P.G. (2002). Continuous distending pressure for respiratory distress syndrome in preterm infants. Cochrane Database Syst. Rev..

[24] Ho J.J., Subramaniam P., Davis P.G. (2020). Continuous positive airway pressure (CPAP) for respiratory distress in preterm infants. Cochrane Database Syst. Rev..

[25] Bamat N., Fierro J., Mukerji A., Wright C.J., Millar D., Kirpalani H. (2021). Nasal continuous positive airway pressure levels for the prevention of morbidity and mortality in preterm infants. Cochrane Database Syst. Rev..

[26] Thomas A.N., Hagan J.L., Lingappan K. (2018). Noninvasive ventilation strategies: Which to choose?. J. Perinatol..

[27] Kribs A., Hummler H. (2016). Ancillary therapies to enhance success of non-invasive modes of respiratory support—Approaches to delivery room use of surfactant and caffeine?. Semin. Neonatal Med..

[28] Cummings J.J., Polin R.A., Committee on Fetus and Newborn, American Academy of Pediatrics (2016). Noninvasive Respiratory Support. Pediatrics.

[29] Verder H., Robertson B., Greisen G., Ebbesen F., Albertsen P., Lundstrom K., Jacobsen T. (1994). Surfactant therapy and nasal continuous positive airway pressure for newborns with respiratory distress syndrome. Danish-Swedish Multicenter Study Group. N. Engl. J. Med..

[30] Kribs A., Pillekamp F., Hünseler C., Vierzig A., Roth B. (2007). Early administration of surfactant in spontaneous breathing with nCPAP: Feasibility and outcome in extremely premature infants (postmenstrual age ≤27 weeks). Paediatr. Anaesth.

[31] Aguar M., Cernada M., Brugada M., Gimeno A., Gutierrez A., Vento M. (2014). Minimally invasive surfactant therapy with a gastric tube is as effective as the intubation, surfactant, and extubation technique in preterm babies. Acta Paediatr..

[32] Kanmaz H.G., Erdeve O., Canpolat F.E., Mutlu B., Dilmen U. (2013). Surfactant administration via thin catheter during spontaneous breathing: Randomized controlled trial. Pediatrics.

[33] Maiwald C.A., Neuberger P., Vochem M., Poets C. (2017). QuickSF: A New Technique in Surfactant Administration. Neonatology.

[34] Dargaville P.A., Aiyappan A., Cornelius A., Williams C., De Paoli A.G. (2011). Preliminary evaluation of a new technique of minimally invasive surfactant therapy. Arch. Dis. Child Fetal Neonatal Ed..

[35] Fabbri L., Klebermass-Schrehof K., Aguar M., Harrison C., Gulczyńska E., Santoro D., Di Castri M., Rigo V. (2018). Five-country manikin study found that neonatologists preferred using the LISAcath rather than the Angiocath for less invasive surfactant administration. Acta Paediatr..

[36] Göpel W., Kribs A., Härtel C., Avenarius S., Teig N., Groneck P., Olbertz D., Roll C., Vochem M., Weller U. (2015). Less invasive surfactant administration is associated with improved pulmonary outcomes in spontaneously breathing preterm infants. Acta Paediatr..

[37] Krajewski P., Chudzik A., Strzałko-Głoskowska B., Górska M., Kmiecik M., Więckowska K., Mesjasz A., Sieroszewski P. (2015). Surfactant administration without intubation in preterm infants with respiratory distress syndrome--our experiences. J. Matern.-Fetal Neonatal Med. Off. J. Eur. Assoc. Perinat. Med. Fed. Asia Ocean. Perinat. Soc. Int. Soc. Perinat. Obstet..

[38] Lau C., Chamberlain R.S., Sun S. (2017). Less Invasive Surfactant Administration Reduces the Need for Mechanical Ventilation in Preterm Infants: A Meta-Analysis. Glob. Pediatr. Health.

[39] Kribs A., Roll C., Göpel W., Wieg C., Groneck P., Laux R., Teig N., Hoehn T., Böhm W., Welzing L. (2015). Nonintubated Surfactant Application vs Conventional Therapy in Extremely Preterm Infants: A Randomized Clinical Trial. JAMA Pediatr..

[40] Härtel C., Paul P., Hanke K., Humberg A., Kribs A., Mehler K., Vochem M., Wieg C., Roll C., Herting E. (2018). Less invasive surfactant administration and complications of preterm birth. Sci. Rep..

